# Supplementary material for: Enhancing drug and cell line representations via contrastive learning for improved anti-cancer drug prioritization
Source: NPJ Precis Oncol. 2024 May 18;8:106. doi: 10.1038/s41698-024-00589-8 (PMC11102516; doi:10.1038/s41698-024-00589-8)
Supplement: Supplementary file 1 — Enhancing drug and cell line representations via contrastive learning for improved anti-cancer drug prioritization supplementary materials [file 41698_2024_589_MOESM1_ESM.docx]

**Supplementary Note 1 KEGG’s documented cancer-related gene list (463).**

ABL1, AGT, AGTR1, AKT1, AKT2, AKT3, ALK, APAF1, APPL1, AR, ARAF, ARNT, ARNT2, BAD, BAK1, BAX, BBC3, BCL2, BCL2L1, BCL2L11, BCR, BDKRB1, BDKRB2, BID, BIRC2, BIRC3, BIRC5, BIRC7, BMP2, BMP4, BRAF, CALM1, CALM2, CALM3, CALML3, CALML4, CALML5, CALML6, CAMK2A, CAMK2B, CAMK2D, CAMK2G, CASP3, CASP7, CASP8, CASP9, CBL, CCDC6, CCNA1, CCNA2, CCND1, CCND2, CCND3, CCNE1, CCNE2, CDC42, CDK2, CDK4, CDK6, CDKN1A, CDKN1B, CDKN2A, CDKN2B, CEBPA, CHUK, CKS1B, CKS2, COL4A1, COL4A2, COL4A3, COL4A4, COL4A5, COL4A6, CREBBP, CRK, CRKL, CSF1R, CSF2RA, CSF2RB, CSF3R, CTBP1, CTBP2, CTNNB1, CUL1, CUL2, CXCL12, CXCL8, CXCR4, CYCS, DAPK1, DAPK2, DAPK3, DCC, DDB2, DLL1, DLL3, DLL4, DVL1, DVL2, DVL3, E2F1, E2F2, E2F3, EDN1, EDNRA, EDNRB, EGF, EGFR, EGLN1, EGLN2, EGLN3, ELK1, ELOB, ELOC, EML4, EP300, EPAS1, EPO, EPOR, ERBB2, ESR1, ESR2, ETS1, F2, F2R, F2RL3, FADD, FAS, FASLG, FGF1, FGF10, FGF16, FGF17, FGF18, FGF19, FGF2, FGF20, FGF21, FGF22, FGF23, FGF3, FGF4, FGF5, FGF6, FGF7, FGF8, FGF9, FGFR1, FGFR2, FGFR3, FGFR4, FLT3, FLT3LG, FLT4, FN1, FOS, FOXO1, FRAT1, FRAT2, FZD1, FZD10, FZD2, FZD3, FZD4, FZD5, FZD6, FZD7, FZD8, FZD9, GADD45A, GADD45B, GADD45G, GLI1, GLI2, GLI3, GNA11, GNAQ, GRB2, GSK3B, GSTA1, GSTA2, GSTA3, GSTA4, GSTA5, GSTM1, GSTM2, GSTM3, GSTM4, GSTM5, GSTO1, GSTO2, GSTP1, GSTT2B, HDAC1, HDAC2, HES1, HES5, HEY1, HEY2, HEYL, HGF, HHIP, HIF1A, HMOX1, HRAS, HSP90AA1, HSP90AB1, HSP90B1, IFNA1, IFNA10, IFNA13, IFNA14, IFNA16, IFNA17, IFNA2, IFNA21, IFNA4, IFNA5, IFNA6, IFNA7, IFNA8, IFNAR1, IFNAR2, IFNG, IFNGR1, IFNGR2, IGF1, IGF1R, IGF2, IKBKB, IKBKG, IL12A, IL12B, IL12RB1, IL12RB2, IL13, IL13RA1, IL15, IL15RA, IL2, IL23A, IL23R, IL2RA, IL2RB, IL2RG, IL3, IL3RA, IL4, IL4R, IL5, IL5RA, IL6, IL6R, IL6ST, IL7, IL7R, ITGA2, ITGA2B, ITGA3, ITGA6, ITGAV, ITGB1, JAG1, JAG2, JAK1, JAK2, JAK3, JUN, JUP, KEAP1, KIF7, KIT, KITLG, KLK3, KNG1, KRAS, LAMA1, LAMA2, LAMA3, LAMA4, LAMA5, LAMB1, LAMB2, LAMB3, LAMB4, LAMC1, LAMC2, LAMC3, LEF1, LPAR1, LPAR2, LPAR3, LPAR4, LPAR5, LPAR6, LRP5, LRP6, MAP2K1, MAP2K2, MAPK1, MAPK10, MAPK3, MAPK8, MAPK9, MAX, MDM2, MECOM, MET, MGST1, MGST2, MGST3, MMP1, MMP2, MMP9, MTOR, MYC, NCOA1, NCOA3, NCOA4, NFE2L2, NFKB1, NFKB2, NFKBIA, NOS2, NOTCH1, NOTCH2, NOTCH3, NOTCH4, NQO1, NRAS, NTRK1, PAX8, PDGFA, PDGFB, PDGFRA, PDGFRB, PGF, PIK3CA, PIK3CB, PIK3CD, PIK3R1, PIK3R2, PIK3R3, PIM1, PIM2, PLCB1, PLCB2, PLCB3, PLCB4, PLCG1, PLCG2, PLD1, PLD2, PMAIP1, PML, POLK, PPARD, PPARG, PRKCA, PRKCB, PRKCG, PTCH1, PTCH2, PTGS2, PTK2, RAC1, RAC2, RAC3, RAF1, RALA, RALB, RALBP1, RALGDS, RARA, RASGRP1, RASGRP2, RASGRP3, RASGRP4, RASSF1, RASSF5, RB1, RBX1, RELA, RET, RHOA, RPS6KA5, RPS6KB1, RPS6KB2, RUNX1, RUNX1T1, RXRA, RXRB, RXRG, SHH, SKP1, SKP2, SLC2A1, SMAD2, SMAD3, SMAD4, SMO, SOS1, SOS2, SP1, SPI1, STAT1, STAT2, STAT3, STAT4, STAT5A, STAT5B, STAT6, STK4, SUFU, TCF7, TCF7L1, TCF7L2, TFG, TGFA, TGFB1, TGFB2, TGFB3, TGFBR1, TGFBR2, TP53, TPM3, TPR, TRAF1, TRAF2, TRAF3, TRAF4, TRAF5, TRAF6, TXNRD1, TXNRD2, TXNRD3, VEGFA, VEGFB, VEGFC, VEGFD, VHL, WNT1, WNT10A, WNT10B, WNT11, WNT16, WNT2, WNT2B, WNT3, WNT3A, WNT4, WNT5A, WNT5B, WNT6, WNT7A, WNT7B, WNT8A, WNT8B, WNT9A, WNT9B, XIAP, ZBTB16, ZBTB17

| **Supplementary Table 1a Cancer-wise cell line counts: pretraining data** | |
| --- | --- |
| Cancer type | Count |
| Bile duct | 29 |
| Bladder | 14 |
| Bone | 28 |
| Brain | 49 |
| Breast | 40 |
| Cervical | 18 |
| Colorectal | 46 |
| Endometrial | 19 |
| Esophageal | 8 |
| Eye | 11 |
| Gastric | 25 |
| Head and Neck | 34 |
| Kidney | 19 |
| Leukemia | 104 |
| Liposarcoma | 10 |
| Liver | 7 |
| Lung | 105 |
| Lymphoma | 83 |
| Myeloma | 30 |
| Neuroblastoma | 26 |
| Ovarian | 34 |
| Pancreatic | 20 |
| Prostate | 8 |
| Rhabdoid | 15 |
| Sarcoma | 29 |
| Skin | 46 |
| Thyroid | 7 |
| Total | 864 |
| Underlined cancer types indicate those which are plotted in **Figure 4** to examine embeddings. | |

| **Supplementary Table 1b Cancer-wise cell line counts: novel cancer test set** | |
| --- | --- |
| Cancer type | Count |
| Bile Duct | 6 |
| Bone | 9 |
| Gallbladder | 1 |
| Gastric | 14 |
| Kidney | 14 |
| Neuroblastoma | 3 |
| Prostate | 2 |
| Rhabdoid | 4 |
| Sarcoma | 6 |
| Thyroid | 8 |
| Total | 67 |

| **Supplementary Table 1c Cancer-wise cell line counts: training folds and trained-on cancer test set** | | | | | | | |
| --- | --- | --- | --- | --- | --- | --- | --- |
| Cancer type | Count | | | | | | |
|  | Fold 1 | Fold 2 | Fold 3 | Fold 4 | Fold 5 | All folds | Testing |
| Bladder | 3 | 3 | 3 | 3 | 5 | 17 | 3 |
| Brain | 5 | 5 | 5 | 5 | 7 | 27 | 5 |
| Breast | 3 | 3 | 3 | 3 | 3 | 15 | 3 |
| Colorectal | 4 | 4 | 4 | 4 | 4 | 20 | 4 |
| Endometrial | 3 | 3 | 3 | 3 | 5 | 17 | 3 |
| Esophageal | 4 | 4 | 4 | 4 | 2 | 18 | 3 |
| Head and Neck | 3 | 3 | 3 | 3 | 5 | 17 | 3 |
| Liver | 3 | 3 | 3 | 3 | 1 | 13 | 2 |
| Lung | 15 | 15 | 15 | 15 | 16 | 76 | 13 |
| Ovarian | 5 | 5 | 5 | 5 | 5 | 25 | 4 |
| Pancreatic | 5 | 5 | 5 | 5 | 5 | 25 | 4 |
| Skin | 6 | 6 | 6 | 6 | 6 | 30 | 5 |
| Total | 59 | 59 | 59 | 59 | 64 | 300 | 52 |

| **Supplementary Table 2 Hyperparameter options: SiamCDR’s drug and cell line encoders (*Enc*) trained by siamese neural networks (SNN).** | |
| --- | --- |
| Hyperparameter | Options tested |
| Number of hidden layers | *1*, **2** |
| Number of units per layer | 16, 32, ***64*** |
| Activation function | ***ReLU***, sigmoid |
| Dropout rate | 0.0, ***0.1***, 0.3 |
| Learning rate | 0.01, ***0.001***, 0.0001 |
| Decay rate | 0.99 |
| Decay steps | 1024 |
| Patience | 10 |
| Minimum delta | 0.0001 |
| Minibatch size | 512 |
| Maximum number of epochs | 1000 |
| For hyperparameters with more than one option, an underlined value, an *italicized value*, or a **bold** value denotes the hyperparameter option with best performing D_embed_ with C_raw_ or *D_embed_ with C_embed_*, or best performing **D_raw_ with** **C_embed_**, respectively. | |

| **Supplementary Table 3a Hyperparameter options:**  **Random forest (RF) classifier.** | |
| --- | --- |
| Hyperparameter | Options tested |
| Criterion | gini, entropy |
| Number of estimators | 10, 25, 50, 100 |
| Minimum samples to split | 5, 10, 20, 25 |
| Underlined values denote the hyperparameter option with best performance. | |

| **Supplementary Table 3b Hyperparameters options:**  **DNN classifier.** | |
| --- | --- |
| Hyperparameter | Options tested |
| Dimensions of hidden layers | 64-32-16; 64-32-8; 64-16-8; 32-16-8; 64-64-64; 32-32-32; 16-16-16; 64-64; 32-32; 16-16; 64-32; 64-16; 32-16; 36; 32; 16 |
| Activation function | ReLU, sigmoid |
| Dropout rate | 0.0, 0.1, 0.3 |
| Learning rate | 0.01, 0.001 |
| Decay rate | 0.99 |
| Decay steps | 50, 500 |
| Patience | 10 |
| Minimum delta | 0.0001 |
| Minibatch size | 256 |
| Maximum epochs | 1000 |
| Underlined values denote the hyperparameter option with best performance. | |

**Supplementary Figure 1 SiamCDR_DNN_ training and validation loss curves.** Solid lines depict the average loss across 5-fold cross validation with shaded regions representing 95% confidence intervals. To examine how training develops when allowed to continue for a greater number of epochs, patience was set to 100.

| **Supplementary Table 4a** $P_{\mathrm{cell}}@k$ **for trained-on cancers.** | | | | | | | | |
| --- | --- | --- | --- | --- | --- | --- | --- | --- |
| Model Architecture | | | $P_{\mathrm{cell}}@k$ | | | | | |
| Drug | Cell line | Classifier | 1 | 2 | 3 | 4 | 5 | 10 |
| **f** | **g** | Logistic  (LR) | *0.8235* | *0.8824* | *0.8824* | *0.8382* | *0.7843* | *0.7000* |
| **e_d_** |  |  | 0.7451 | 0.6471 | 0.5621 | 0.4902 | 0.4588 | 0.3667 |
| **f** | **e_c_** |  | 0.9412 | 0.9112 | 0.8693 | 0.8593 | 0.8275 | 0.8077 |
| **e_d_** |  |  | 0.9412 | 0.9020 | 0.8497 | 0.8480 | 0.8275 | 0.8128 |
| **f** | **g** | Random Forest (RF) | *0.9412* | *0.8922* | *0.9020* | *0.8676* | *0.8392* | *0.8385* |
| **e_d_** |  |  | 0.8039 | 0.8529 | 0.8366 | 0.8333 | 0.8118 | 0.7718 |
| **f** | **e_c_** |  | 0.9412 | 0.9020 | 0.8824 | 0.8676 | **0.8588** | 0.8103 |
| **e_d_** |  |  | 0.9608 | **0.9412** | **0.9085** | **0.8775** | 0.8549 | 0.8026 |
| **f** | **g** | DNN | *0.9412* | *0.9020* | *0.8889* | *0.8578* | *0.8471* | *0.8308* |
| **e_d_** |  |  | 0.8235 | 0.6471 | 0.5686 | 0.5086 | 0.4784 | 0.4282 |
| **f** | **e_c_** |  | **0.9804** | **0.9412** | 0.8654 | 0.8529 | 0.8235 | **0.8436** |
| **e_d_** |  |  | **0.9804** | 0.9216 | 0.9020 | 0.8529 | 0.8471 | 0.8385 |
| For model architecture, underlined parameters denote highest performing model for that classifier. **Bolded values** denote top performance for that metric across all model variations; underlined values denote top performance for that metric across all variations of a given end classifier (LM, RF, DNN); and *italicized* *values* denote a classifier’s vanilla performance without any feature embedding. | | | | | | | | |

| **Supplementary Table 4b** $P_{\mathrm{cell}}@k$ **for novel cancers.** | | | | | | | | |
| --- | --- | --- | --- | --- | --- | --- | --- | --- |
| Model Architecture | | | $P_{\mathrm{cell}}@k$ | | | | | |
| Drug | Cell line | Classifier | 1 | 2 | 3 | 4 | 5 | 10 |
| **f** | **g** | Logistic  (LR) | *0.8923* | *0.9154* | *0.8615* | *0.8423* | *0.8000* | *0.7157* |
| **e_d_** |  |  | 0.7385 | 0.6615 | 0.6000 | 0.5231 | 0.4954 | 0.4294 |
| **f** | **e_c_** |  | 0.9538 | 0.9077 | **0.9077** | 0.8731 | 0.8585 | 0.8020 |
| **e_d_** |  |  | 0.9538 | 0.9077 | 0.9026 | 0.8731 | 0.8615 | 0.8078 |
| **f** | **g** | Random Forest (RF) | *0.9538* | *0.9154* | *0.8821* | *0.8654* | *0.8554* | *0.8235* |
| **e_d_** |  |  | 0.8308 | 0.8538 | 0.8462 | 0.8385 | 0.8154 | 0.7843 |
| **f** | **e_c_** |  | **0.9846** | 0.9385 | 0.8718 | 0.8423 | 0.8185 | 0.7922 |
| **e_d_** |  |  | 0.9538 | **0.9538** | 0.8923 | 0.8500 | 0.8277 | 0.7941 |
| **f** | **g** | DNN | *0.9692* | *0.9231* | *0.9026* | ***0.8885*** | *0.8585* | *0.8157* |
| **e_d_** |  |  | 0.7692 | 0.6538 | 0.6103 | 0.5385 | 0.5077 | 0.4765 |
| **f** | **e_c_** |  | **0.9846** | 0.9462 | 0.9026 | 0.8692 | 0.8615 | 0.8275 |
| **e_d_** |  |  | 0.9538 | 0.9154 | 0.8923 | 0.8769 | **0.8738** | 0.8216 |
| **Bolded values** denote top performance for that metric across all model variations; underlined values denote top performance for that metric across all variations of a given end classifier (LM, RF, DNN); and *italicized* *values* denote a classifier’s vanilla performance without any feature embedding. | | | | | | | | |

| **Supplementary Table 5a** $P_{\mathrm{cancer}}@k$ **for trained-on cancers** | | | | | | | |
| --- | --- | --- | --- | --- | --- | --- | --- |
| Model Architecture | | | $P_{\mathrm{cancer}}@k$ | | | | |
| Drug | Cell line | Classifier | 1 | 2 | 3 | 4 | 5 |
| **f** | **g** | Logistic  (LR) | *0.8419* | *0.8862* | *0.8912* | *0.8526* | *0.8019* |
| **e_d_** |  |  | 0.7311 | 0.6386 | 0.5554 | 0.4913 | 0.4564 |
| **f** | **e_c_** |  | 0.9450 | 0.9265 | 0.8824 | 0.8564 | 0.8280 |
| **e_d_** |  |  | 0.9450 | 0.9161 | 0.8569 | 0.8505 | 0.8280 |
| **f** | **g** | Random Forest (RF) | *0.9450* | *0.9043* | *0.9109* | *0.8838* | *0.8484* |
| **e_d_** |  |  | 0.8363 | 0.8612 | 0.8540 | 0.8420 | 0.8137 |
| **f** | **e_c_** |  | 0.9663 | 0.9288 | 0.8916 | 0.8713 | **0.8606** |
| **e_d_** |  |  | 0.9728 | **0.9591** | **0.9171** | 0.8850 | 0.8571 |
| **f** | **g** | DNN | *0.9450* | *0.9160* | *0.8933* | *0.8600* | *0.8453* |
| **e_d_** |  |  | 0.8283 | 0.6427 | 0.5647 | 0.5016 | 0.4814 |
| **f** | **e_c_** |  | **0.9792** | 0.9378 | 0.9040 | 0.8663 | 0.8327 |
| **e_d_** |  |  | **0.9792** | 0.9297 | 0.9109 | 0.8593 | 0.8517 |
| **Bolded values** denote top performance for that metric across all model variations; underlined values denote top performance for that metric across all variations of a given end classifier (LM, RF, DNN); and *italicized* *values* denote a classifier’s vanilla performance without any feature embedding. | | | | | | | |

| **Supplementary Table 5b** $P_{\mathrm{cancer}}@k$ **for novel cancers.** | | | | | | | |
| --- | --- | --- | --- | --- | --- | --- | --- |
| Model Architecture | | | $P_{\mathrm{cancer}}@k$ | | | | |
| Drug | Cell line | Classifier | 1 | 2 | 3 | 4 | 5 |
| **f** | **g** | Logistic  (LR) | *0.9290* | *0.9361* | *0.8597* | *0.8204* | *0.7886* |
| **e_d_** |  |  | 0.6638 | 0.6544 | 0.5808 | 0.5053 | 0.4730 |
| **f** | **e_c_** |  | 0.9646 | 0.9222 | **0.9289** | 0.8861 | 0.8485 |
| **e_d_** |  |  | 0.9646 | 0.9222 | 0.9263 | 0.8861 | 0.8552 |
| **f** | **g** | Random Forest (RF) | *0.9721* | *0.9460* | *0.8904* | *0.8605* | *0.8609* |
| **e_d_** |  |  | 0.8106 | 0.8524 | 0.8552 | 0.8284 | 0.7919 |
| **f** | **e_c_** |  | **0.9923** | 0.9505 | 0.8544 | 0.8315 | 0.8068 |
| **e_d_** |  |  | 0.9769 | **0.9641** | 0.8820 | 0.8856 | 0.8187 |
| **f** | **g** | DNN | *0.9846* | *0.9347* | *0.9239* | ***0.8953*** | ***0.8663*** |
| **e_d_** |  |  | 0.7710 | 0.6461 | 0.6224 | 0.5390 | 0.5130 |
| **f** | **e_c_** |  | **0.9923** | 0.9542 | 0.9064 | 0.8573 | 0.8290 |
| **e_d_** |  |  | 0.9769 | 0.9285 | 0.8764 | 0.8706 | 0.8582 |
| **Bolded values** denote top performance for that metric across all model variations; underlined values denote top performance for that metric across all variations of a given end classifier (LM, RF, DNN); and *italicized* *values* denote a classifier’s vanilla performance without any feature embedding. | | | | | | | |

| **Supplementary Table 6a DeepDSC’s** $P_{\mathrm{cancer}}@k$ **for trained­‑on cancers.** | | | | | | |
| --- | --- | --- | --- | --- | --- | --- |
| Cancer type | $P_{\mathrm{cancer}}@1$ | $P_{\mathrm{cancer}}@2$ | $P_{\mathrm{cancer}}@3$ | $P_{\mathrm{cancer}}@4$ | $P_{\mathrm{cancer}}@5$ |  |
| Bladder | 1.0000 | 0.8333 | 0.8889 | 0.8333 | 0.7333 |  |
| Brain | 0.6667 | 0.8333 | 0.5556 | 0.5833 | 0.6000 |  |
| Breast | 1.0000 | 0.8333 | 0.5556 | 0.6667 | 0.5333 |  |
| Colorectal | 1.0000 | 0.8750 | 0.7500 | 0.6250 | 0.5500 |  |
| Endometrial | 1.0000 | 0.8333 | 0.7778 | 0.7500 | 0.6667 |  |
| Esophageal | 1.0000 | 1.0000 | 0.8333 | 0.7500 | 0.7000 |  |
| Head and Neck | 1.0000 | 1.0000 | 0.6667 | 0.5833 | 0.6000 |  |
| Liver | 1.0000 | 1.0000 | 0.7556 | 0.7500 | 0.6000 |  |
| Lung | 0.9333 | 0.9000 | 0.6667 | 0.7333 | 0.6667 |  |
| Ovarian | 0.6000 | 0.8000 | 0.6667 | 0.7000 | 0.6400 |  |
| Pancreatic | 0.8000 | 0.8000 | 0.6667 | 0.7000 | 0.6800 |  |
| Skin | 1.0000 | 0.8333 | 0.6667 | 0.5833 | 0.5333 |  |
| Overall | 0.9167 | 0.8785 | 0.7227 | 0.6882 | 0.6253 |  |

| **Supplementary Table 6b SiamCDR_RF_’s** $P_{\mathrm{cancer}}@k$ **for trained­‑on cancers.** | | | | | |
| --- | --- | --- | --- | --- | --- |
| Cancer type | $P_{\mathrm{cancer}}@1$ | $P_{\mathrm{cancer}}@2$ | $P_{\mathrm{cancer}}@3$ | $P_{\mathrm{cancer}}@4$ | $P_{\mathrm{cancer}}@5$ |
| Bladder | 1.0000 | 1.0000 | 1.0000 | 0.9167 | 0.9333 |
| Brain | 1.0000 | 0.8750 | 0.8333 | 0.8125 | 0.7500 |
| Breast | 1.0000 | 1.0000 | 0.7778 | 0.7500 | 0.8000 |
| Colorectal | 1.0000 | 1.0000 | 1.0000 | 0.8750 | 0.9000 |
| Endometrial | 1.0000 | 1.0000 | 1.0000 | 0.9167 | 0.8667 |
| Esophageal | 1.0000 | 0.8333 | 0.7778 | 0.7500 | 0.8000 |
| Head and Neck | 1.0000 | 1.0000 | 1.0000 | 1.0000 | 0.9333 |
| Liver | 1.0000 | 1.0000 | 1.0000 | 1.0000 | 0.9000 |
| Lung | 0.9231 | 0.9231 | 0.8974 | 0.8846 | 0.8615 |
| Ovarian | 1.0000 | 1.0000 | 1.0000 | 0.8750 | 0.8615 |
| Pancreatic | 0.7500 | 0.8750 | 0.8333 | 0.8125 | 0.8000 |
| Skin | 1.0000 | 1.0000 | 0.8667 | 0.9000 | 0.8000 |
| Overall | 0.9728 | 0.9589 | 0.9155 | 0.8744 | 0.8454 |

| **Supplementary Table 6c SiamCDR_LR_’s** $P_{\mathrm{cancer}}@k$ **for trained­‑on cancers.** | | | | | | |
| --- | --- | --- | --- | --- | --- | --- |
| Cancer type | $P_{\mathrm{cancer}}@1$ | $P_{\mathrm{cancer}}@2$ | $P_{\mathrm{cancer}}@3$ | $P_{\mathrm{cancer}}@4$ | $P_{\mathrm{cancer}}@5$ |  |
| Bladder | 1.0000 | 1.0000 | 1.0000 | 1.0000 | 1.0000 |  |
| Brain | 1.0000 | 0.8750 | 0.8333 | 0.7500 | 0.7500 |  |
| Breast | 1.0000 | 1.0000 | 0.7778 | 0.8333 | 0.8000 |  |
| Colorectal | 1.0000 | 1.0000 | 0.9167 | 0.9375 | 0.9000 |  |
| Endometrial | 1.0000 | 1.0000 | 0.8889 | 0.9167 | 0.8667 |  |
| Esophageal | 0.6667 | 0.8333 | 0.8889 | 0.7500 | 0.8000 |  |
| Head and Neck | 1.0000 | 1.0000 | 0.8889 | 0.8333 | 0.8000 |  |
| Liver | 1.0000 | 1.0000 | 1.0000 | 1.0000 | 1.0000 |  |
| Lung | 0.9231 | 0.8846 | 0.8718 | 0.9038 | 0.8462 |  |
| Ovarian | 1.0000 | 0.8750 | 0.8333 | 0.8125 | 0.8500 |  |
| Pancreatic | 0.7500 | 0.7500 | 0.8333 | 0.7500 | 0.7500 |  |
| Skin | 1.0000 | 0.9000 | 0.8000 | 0.8500 | 0.8000 |  |
| Overall | 0.9450 | 0.9265 | 0.8777 | 0.8614 | 0.8469 |  |

| **Supplementary Table 6d SiamCDR_DNN_’s** $P_{\mathrm{cancer}}@k$ **for trained­‑on cancers.** | | | | | |
| --- | --- | --- | --- | --- | --- |
| Cancer type | $P_{\mathrm{cancer}}@1$ | $P_{\mathrm{cancer}}@2$ | $P_{\mathrm{cancer}}@3$ | $P_{\mathrm{cancer}}@4$ | $P_{\mathrm{cancer}}@5$ |
| Bladder | 1.0000 | 1.0000 | 1.0000 | 1.0000 | 0.9333 |
| Brain | 1.0000 | 0.8750 | 0.8333 | 0.8125 | 0.7500 |
| Breast | 1.0000 | 0.8333 | 0.8889 | 0.8333 | 0.8667 |
| Colorectal | 1.0000 | 1.0000 | 0.9167 | 0.9375 | 0.8500 |
| Endometrial | 1.0000 | 1.0000 | 0.8889 | 0.9167 | 0.8667 |
| Esophageal | 1.0000 | 0.8333 | 0.8889 | 0.8333 | 0.7333 |
| Head and Neck | 1.0000 | 1.0000 | 1.0000 | 0.9167 | 0.9333 |
| Liver | 1.0000 | 1.0000 | 1.0000 | 1.0000 | 1.0000 |
| Lung | 0.9231 | 0.8462 | 0.8974 | 0.8654 | 0.8769 |
| Ovarian | 1.0000 | 1.0000 | 0.9167 | 0.9375 | 0.8000 |
| Pancreatic | 0.7500 | 0.7500 | 0.8333 | 0.8125 | 0.7500 |
| Skin | 1.0000 | 1.0000 | 0.8667 | 0.8500 | 0.8000 |
| Overall | 0.9728 | 0.9282 | 0.9109 | 0.8929 | 0.8467 |

| **Supplementary Table 7a DeepDSC’s** $P_{\mathrm{cancer}}@k$ **for novel cancer types.** | | | | | |
| --- | --- | --- | --- | --- | --- |
| Cancer type | $P_{\mathrm{cancer}}@1$ | $P_{\mathrm{cancer}}@2$ | $P_{\mathrm{cancer}}@3$ | $P_{\mathrm{cancer}}@4$ | $P_{\mathrm{cancer}}@5$ |
| Bile | 0.6000 | 0.7000 | 0.6667 | 0.5550 | 0.4400 |
| Bone | 1.0000 | 0.8889 | 0.7407 | 0.6667 | 0.6444 |
| Gallbladder | 1.0000 | 1.0000 | 0.6667 | 0.5000 | 0.4000 |
| Gastric | 1.0000 | 0.9286 | 0.8095 | 0.7857 | 0.7429 |
| Kidney | 0.6154 | 0.6583 | 0.6923 | 0.6154 | 0.5846 |
| Neuroblastoma | 1.0000 | 1.0000 | 0.8889 | 0.9167 | 0.9333 |
| Prostate | 1.0000 | 1.0000 | 0.8333 | 0.8750 | 0.7000 |
| Rhabdoid | 0.7500 | 0.7500 | 0.6667 | 0.6875 | 0.6500 |
| Sarcoma | 0.8333 | 0.8333 | 0.7778 | 0.7083 | 0.6000 |
| Thyroid | 0.7500 | 0.8125 | 0.6667 | 0.7188 | 0.6750 |
| Overall | 0.8549 | 0.8567 | 0.7409 | 0.7024 | 0.6370 |

| **Supplementary Table 7b SiamCDR_RF_’s** $P_{\mathrm{cancer}}@k$ **for novel cancer types.** | | | | | |
| --- | --- | --- | --- | --- | --- |
| Cancer type | $P_{\mathrm{cancer}}@1$ | $P_{\mathrm{cancer}}@2$ | $P_{\mathrm{cancer}}@3$ | $P_{\mathrm{cancer}}@4$ | $P_{\mathrm{cancer}}@5$ |
| Bile | 1.0000 | 0.9000 | 0.9333 | 0.9000 | 0.8800 |
| Bone | 1.0000 | 1.0000 | 0.9630 | 0.9167 | 0.8889 |
| Gallbladder | 1.0000 | 1.0000 | 1.0000 | 0.7500 | 0.6000 |
| Gastric | 1.0000 | 0.9643 | 0.9524 | 0.9286 | 0.9286 |
| Kidney | 0.7692 | 0.7692 | 0.7179 | 0.6923 | 0.6769 |
| Neuroblastoma | 1.0000 | 0.8333 | 0.7778 | 0.6667 | 0.7333 |
| Prostate | 1.0000 | 1.0000 | 1.0000 | 1.0000 | 1.0000 |
| Rhabdoid | 1.0000 | 1.0000 | 0.9167 | 0.8750 | 0.9000 |
| Sarcoma | 0.8333 | 0.9167 | 0.9444 | 0.8750 | 0.8667 |
| Thyroid | 1.0000 | 0.9375 | 0.9583 | 0.9062 | 0.8500 |
| Overall | 0.9603 | 0.9321 | 0.9164 | 0.8510 | 0.8324 |

| **Supplementary Table 7c SiamCDR_LR_’s** $P_{\mathrm{cancer}}@k$ **for novel cancer types.** | | | | | |
| --- | --- | --- | --- | --- | --- |
| Cancer type | $P_{\mathrm{cancer}}@1$ | $P_{\mathrm{cancer}}@2$ | $P_{\mathrm{cancer}}@3$ | $P_{\mathrm{cancer}}@4$ | $P_{\mathrm{cancer}}@5$ |
| Bile Duct | 0.8000 | 0.8000 | 0.8667 | 0.8500 | 0.8000 |
| Bone | 1.0000 | 0.9444 | 0.8889 | 0.8611 | 0.8667 |
| Gallbladder | 1.0000 | 1.0000 | 1.0000 | 0.7500 | 0.6000 |
| Gastric | 1.0000 | 1.0000 | 1.0000 | 0.9643 | 0.9429 |
| Kidney | 0.8462 | 0.7692 | 0.7436 | 0.7308 | 0.7077 |
| Neuroblastoma | 1.0000 | 0.8333 | 0.8889 | 0.9167 | 0.9333 |
| Prostate | 1.0000 | 1.0000 | 1.0000 | 1.0000 | 0.9000 |
| Rhabdoid | 1.0000 | 1.0000 | 1.0000 | 0.9375 | 0.9500 |
| Sarcoma | 1.0000 | 1.0000 | 1.0000 | 1.0000 | 0.9333 |
| Thyroid | 1.0000 | 0.9375 | 0.9167 | 0.9062 | 0.9000 |
| Overall | 0.9646 | 0.9285 | 0.9305 | 0.8917 | 0.8534 |

| **Supplementary Table 7d SiamCDR_DNN_’s** $P_{\mathrm{cancer}}@k$ **for novel cancer types.** | | | | | |
| --- | --- | --- | --- | --- | --- |
| Cancer type | $P_{\mathrm{cancer}}@1$ | $P_{\mathrm{cancer}}@2$ | $P_{\mathrm{cancer}}@3$ | $P_{\mathrm{cancer}}@4$ | $P_{\mathrm{cancer}}@5$ |
| Bile Duct | 1.0000 | 0.9000 | 0.8667 | 0.8500 | 0.8800 |
| Bone | 1.0000 | 0.9444 | 0.9259 | 0.8889 | 0.9111 |
| Gallbladder | 1.0000 | 1.0000 | 1.0000 | 0.7500 | 0.8000 |
| Gastric | 1.0000 | 1.0000 | 0.9524 | 0.9464 | 0.9142 |
| Kidney | 0.9231 | 0.8077 | 0.7949 | 0.7500 | 0.7231 |
| Neuroblastoma | 1.0000 | 1.0000 | 1.0000 | 0.8333 | 0.8000 |
| Prostate | 1.0000 | 1.0000 | 1.0000 | 1.0000 | 0.9000 |
| Rhabdoid | 1.0000 | 1.0000 | 0.9167 | 0.9375 | 0.9000 |
| Sarcoma | 0.8333 | 0.9167 | 0.9444 | 0.9167 | 0.8667 |
| Thyroid | 1.0000 | 1.0000 | 0.9583 | 0.9375 | 0.8500 |
| Overall | 0.9756 | 0.9569 | 0.9359 | 0.8810 | 0.8545 |

| **Supplementary Table 8 Mechanisms of action (MOA) with at least 10 drugs in pretraining data.** | |
| --- | --- |
| MOA | Count |
| EGFR inhibitor | 32 |
| HDAC inhibitor | 25 |
| Glucocorticoid receptor agonist | 24 |
| Topoisomerase inhibitor | 23 |
| Tubulin polymerization inhibitor | 21 |
| Aurora kinase inhibitor | 18 |
| Histamine receptor antagonist | 18 |
| MEK inhibitor | 18 |
| Adrenergic receptor antagonist | 17 |
| PI3K inhibitor | 16 |
| Protein synthesis inhibitor | 16 |
| mTOR inhibitor | 16 |
| CDK inhibitor | 16 |
| Cyclooxygenase inhibitor | 15 |
| Adrenergic receptor agonist | 14 |
| Dopamine receptor antagonist | 13 |
| Glutamate receptor antagonist | 13 |
| HSP inhibitor | 13 |
| Sodium channel blocker | 11 |
| Benzodiazepine receptor agonist | 11 |
| Phosphodiesterase inhibitor | 10 |
| DNA inhibitor | 10 |
| Total | 370 |

| **Supplementary Table 9 Data, hardware, and major software used.** | | |
| --- | --- | --- |
| Data set | Source (DOI) | Version |
| PRISM repurposing secondary screen | https://doi.org/10.1038/s43018-019-0018-6 | PRISM Repurposing 19Q4 |
| Cell line gene expression and cancer types | https://doi.org/10.1038/s41586-019-1186-3 | DepMap Public 22Q2 |
| Hardware | Source | Version |
| CPU | Ohio Supercomputer Center | Intel Xeon 8268s Cascade Lakes |
| GPU | Ohio Supercomputer Center | NVIDIA Volta V100 |
| Software | Version / DOI | |
| Python | 3.8.12 | |
| Tensorflow | 2.8.0 | |
| Scikit-learn | 1.0.2 | |
| DeepDSC | 10.1109/TCBB.2019.2919581 | |

**Supplementary Note 2 Comparing the performances of SiamCDR_RF_ and SubCDR.**

We compare SiamCDR_RF_ against a recently published state-of-the-art: SubCDR. This was done to ascertain whether SiamCDR_RF_’s unsupervised learning approach is better able to capture the information and patterns relevant to predicting CDR compared to the hardcoded annotations used by SubCDR. Specifically, we evaluate both models’ performance on $P_{\text{c}\mathrm{ell}}@k=[1, 2, 3, 4, 5]$ and $P_{\text{c}\mathrm{ancer}}@k=[1, 2, 3, 4, 5]$. SubCDR masks out z-score normalized gene expression values of genes not involved in a cell tumorigenesis using tumor type-specific gene annotations. For its drug representations, SubCDR decomposes molecules into their subcomponents using the BRICS algorithm. Interestingly, ‘side information’ is also supplied as input to the model, is obtained through matrix factorization of known CDR values.

To compare performance, we first retrained both models using a subset of the curated data provided by SubCDR. Specifically, we first filtered cell lines to obtain the intersection of their dataset and our own, yielding 588 cell lines. This was done for three reasons. First, SubCDR leverages z-score normalized gene expression values. Given that our cell line encoder was pre-trained on log2 transformed gene expression counts (TPM), we needed to pretrain a new encoder using z-scores. By finding the intersection of cell lines, we were able to calculate z-scores for our entire data set and subsequently pretrain our cell line encoder on approximately 1,000 cell lines not present in SubCDR’s data set. Secondly, by retaining a subset of SubCDR’s original data for training, we would ensure that we had identical gene annotations used to originally train SubCDR. Finally, by using cell lines that were in our data set too, we would ensure that we would have the cancer type labels necessary for calculating $P_{\text{c}\mathrm{ancer}}@k$. Note that there were nine genes used by SubCDR that were not sequenced in our dataset. These genes were excluded, yielding a total of 647 genes. To minimize the inconsistency of information being input to each model, we also retrained our drug encoder using 512-bit Morgan fingerprints (radius=2) to represent each drug as this is the representation used for SubCDR’s decomposed molecular subcomponents.

It is worth noting that the training protocol described in SubCDR manuscript does not stratify drug-cell line pairs across training and testing subsets. That is, while the specific drug-cell line pairs are distinct between training and testing, each cell line and drug in the training set will also be encountered during testing. This approach can evaluate how well a model predicts cellular response to new combinations of known drugs and cell lines. However, without stratification, you cannot evaluate how your model generalizes to new drugs and new cell lines which would be more representative of a real-world use case. For example, a physician may want to ascertain which drugs are good candidates for treating a new patient’s cancer. To compare performance on this more relevant task, we train both SiamCDR_RF_ and SubCDR on subset of cell lines that were sampled from each cancer type. This is done such that 10% of cell lines or one cell line of a given cancer type are/is retained for testing, whichever is larger.

SubCDR also requires all cell lines and drugs to be in the training data when it creates its ‘side information’. Inference cannot be done for any cell line or drug not included as there will be no ‘side information’ available. Given the stratified training scheme outlined above, there are two options to support inference over cell lines withheld during training. First, CDR values may be randomly sampled for each cell line in the testing data to include when producing the ‘side information’. This is problematic as it introduces data leakage. That is, the labels used during testing will be supplied to the model as input during training. Another option is that the withheld cell lines can be added to the CDR matrix for factorization with a mask over all their CDR values. Because this secondary approach essentially removes the ‘side information’ for each withheld cell line, it is likely to result in a substantial reduction in performance compared to the method that introduces data leakage. However, the complete masking of CDR information would be more representative of SubCDR’s would perform on new cell lines. We train two versions of SubCDR using both approaches for creating SubCDR’s ‘side information’ to compare how they affect performance relative to SiamCDR_RF_.

The test performance of all three trained models with respect to $P_{\text{c}\mathrm{ell}}@k=[1, 2, 3, 4, 5]$ and $P_{\text{c}\mathrm{ancer}}@k=[1, 2, 3, 4, 5]$ is presented in **Supplementary Tables 10** and **11**, respectively. Two-tailed independent t-tests with Bonferroni multiple hypothesis correction are conducted to evaluate how significant the difference in performance was between SiamCDR_RF_ and SubCDR trained with and without data leakage (SubCDR_leak_ and SubCDR_noLeak_, respectively). We observe that SiamCDR_RF_ outperforms both versions of SubCDR for both $P_{\text{c}\mathrm{ell}}@k=1$ and $P_{\text{c}\mathrm{ancer}}@k=1$. SubCDR_leak_ performs better than SiamCDR_RF_ for $P_{\text{c}\mathrm{ell}}@k>1$ and $P_{\text{c}\mathrm{ancer}}@k>1$. However, this difference is not significant. SiamCDR_RF_ does significantly outperform SubCDR_noLeak_ for all measures. This result highlights the utility of SiamCDR_RF_’s unsupervised representation learning strategy as it performs as well as if not better than SubCDR when it was trained with the test labels. It also further demonstrates SiamCDR_RF_’s strength at generalizing what it has learned to produce viable therapeutic candidates for unseen cell lines.

| **Supplementary Tables 10** $P_{\text{cell}}@k$ **for SiamCDR_RF_ and SubCDR.** | | | | | | |
| --- | --- | --- | --- | --- | --- | --- |
| Model | | $P_{\text{cell}}@1$ | $P_{\text{cell}}@2$ | $P_{\text{cell}}@3$ | $P_{\text{cell}}@4$ | $P_{\text{cell}}@5$ |
| SiamCDR_RF_ | | **0.9800** | 0.9200 | 0.8867 | 0.8700 | 0.8520 |
| SubCDR | *with leakage* | 0.9600 | **0.9400** | **0.9200** | **0.8950** | **0.8720** |
|  | *no leakage* | ***0.6800 | ***0.6800 | ***0.6733 | ***0.6700 | ***0.6480 |
| Significance levels ($\alpha\leq0.1, 0.05, 0.01$; indicated by *, **, ***, respectively) are determined from *p-*values obtained via Bonferroni correction (n=2) of two-tailed t-tests comparing the performances of SiamCDR_RF_ against SubCDR with and without data leakage (SubCDR_leak_ and SubCDR_noLeak_). | | | | | | |

| **Supplementary Tables 11** $P_{\text{cancer}}@k$ **for SiamCDR_RF_ and SubCDR.** | | | | | | |
| --- | --- | --- | --- | --- | --- | --- |
| Model | | $P_{\text{cell}}@1$ | $P_{\text{cell}}@2$ | $P_{\text{cell}}@3$ | $P_{\text{cell}}@4$ | $P_{\text{cell}}@5$ |
| SiamCDR_RF_ | | **0.9800** | 0.9015 | 0.8499 | 0.8241 | 0.7965 |
| SubCDR | *with leakage* | 0.9400 | **0.9183** | **0.8978** | **0.8575** | **0.8313** |
|  | *no leakage* | ***0.5794 | ***0.5794 | ***0.6038 | ***0.5995 | ***0.5774 |
| Significance levels ($\alpha\leq0.1, 0.05, 0.01$; indicated by *, **, ***, respectively) are determined from *p*‑values obtained via Bonferroni correction (n=2) of two‑tailed t‑tests comparing the performances of SiamCDR_RF_ against SubCDR with and without data leakage (SubCDR_leak_ and SubCDR_noLeak_). | | | | | | |

**Supplementary Note 3 Comparing learned cell line embeddings produced by Siamese neural networks and momentum contrastive learning.** We also compare the efficacy of our proposed Siamese neural network framework against another popular few-shot learning strategy: momentum contrastive learning (MoCo v3). Specifically, we evaluate how well the embeddings produced by each method are able to differentiate cell lines with respect to their cancer type. Conventionally, MoCo is implemented with the goal of improving model differentiation of unique. This is done by leveraging random augmentations performed before applying the query and key encoders enables an infinite number of image variations to be produced for a single image. Given this context, rather than augmenting the transcriptomic profile of each cell line, as is typically done in MoCo, each cell line is considered to be an augmentation of its respective cancer type. Each transcriptomic profile was filtered to retain only the genes in the subset proposed by the authors of SubCDR. This was done to mitigate our own biases.

We find that our Siamese framework has similar cohesion (intra-cancer similarity) to the MoCo v3 framework but achieves nearly 5-times greater separation between cancer types ($p<0.001$). See **Supplementary Figure 2** for a qualitative depiction of the differences in cluster quality. We use the same approach as was described in the ‘**Clustering**’ subsection of Methods. We observe distinct clusters for each cancer type when using the embeddings produced by the Siamese framework (**Supplementary Figure 2b**). Conversely, there does not appear to be any meaningful organization of cell lines when they are clustered using the embeddings learned within the MoCo v3 framework. It is likely that our deviation from its typical implementation may explain MoCo’s reduced performance. In doing so, this method enables comparison against a larger number of negative samples simultaneously using the InfoNCE loss function. However, by treating pairs of cell lines from a given cancer type as augmented versions of the cancer type, we are forced to limit our maximum minibatch size to the number of cancer types present in our dataset, reducing the learning efficiency of the MoCo method.


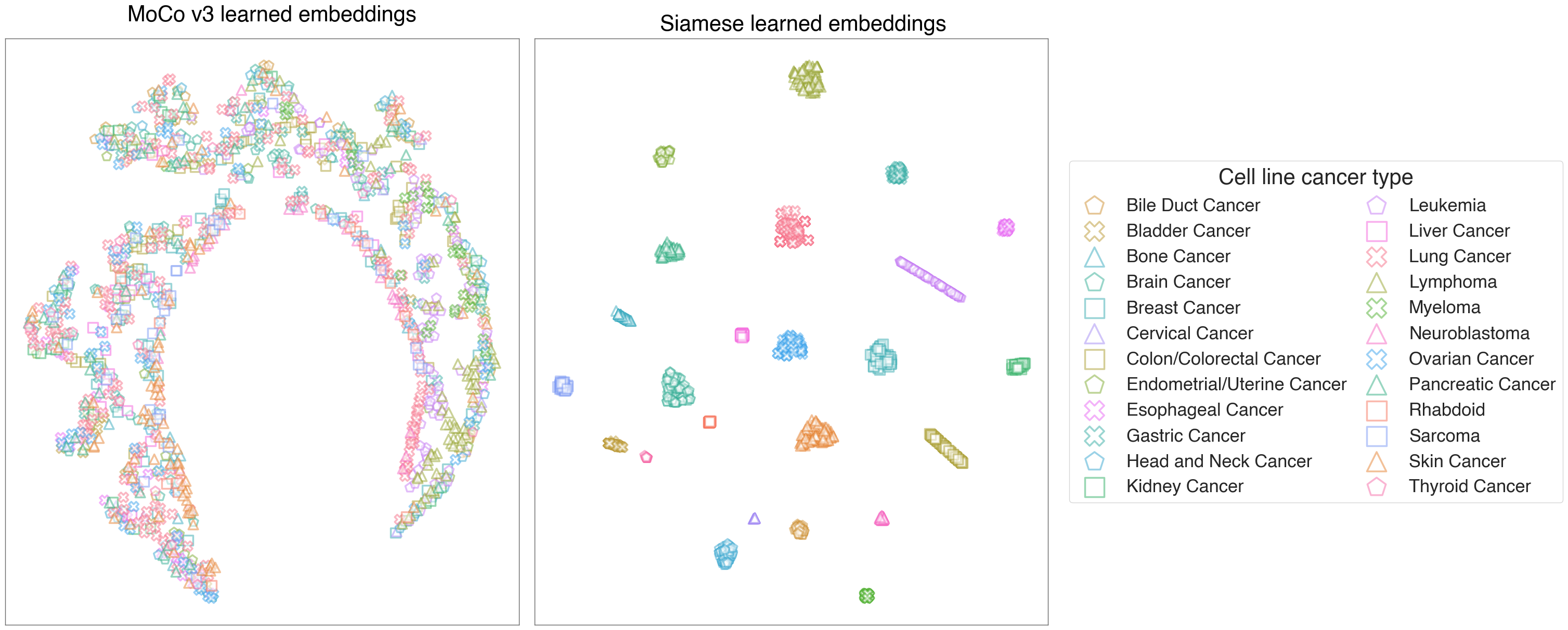


**Supplementary Figure 2 t-SNE plots of cell line representations learned by MoCo v3 (a) or Siamese neural network (b) frameworks.** Each marked represents a distinct cell line with cancer type being denoted with a unique shape-color combination (see plot legend).
