# Supplementary material for: Enhancing drug and cell line representations via contrastive learning for improved anti-cancer drug prioritization
Source: NPJ Precis Oncol. 2024 May 18;8:106. doi: 10.1038/s41698-024-00589-8 (PMC11102516; doi:10.1038/s41698-024-00589-8)
Supplement: Supplementary file 2 — Reporting Summary [file 41698_2024_589_MOESM2_ESM.pdf]

nature portfolio

Corresponding author(s): Dr Ning

Last updated by author(s): Feb 23, 2024

Reporting Summary

Nature Portfolio wishes to improve the reproducibility of the work that we publish. This form provides structure for consistency and transparency in reporting. For further information on Nature Portfolio policies, see our Editorial Policies and the Editorial Policy Checklist.

Statistics

For all statistical analyses, confirm that the following items are present in the figure legend, table legend, main text, or Methods section.

n/a	Confirmed	
☐	☒	The exact sample size (*n*) for each experimental group/condition, given as a discrete number and unit of measurement
☐	☒	A statement on whether measurements were taken from distinct samples or whether the same sample was measured repeatedly
☐	☒	The statistical test(s) used AND whether they are one- or two-sided<br>*Only common tests should be described solely by name; describe more complex techniques in the Methods section.*
☒	☐	A description of all covariates tested
☐	☒	A description of any assumptions or corrections, such as tests of normality and adjustment for multiple comparisons
☐	☒	A full description of the statistical parameters including central tendency (e.g. means) or other basic estimates (e.g. regression coefficient) AND variation (e.g. standard deviation) or associated estimates of uncertainty (e.g. confidence intervals)
☐	☒	For null hypothesis testing, the test statistic (e.g. F, t, r) with confidence intervals, effect sizes, degrees of freedom and P value noted<br>*Give P values as exact values whenever suitable.*
☒	☐	For Bayesian analysis, information on the choice of priors and Markov chain Monte Carlo settings
☒	☐	For hierarchical and complex designs, identification of the appropriate level for tests and full reporting of outcomes
☒	☐	Estimates of effect sizes (e.g. Cohen's *d*, Pearson's *r*), indicating how they were calculated

Our web collection on statistics for biologists contains articles on many of the points above.

Software and code

Policy information about availability of computer code

Data collection	No code was used for data collection in this study
Data analysis	The code used to produce our model is freely available here (https://github.com/ninglab/SiamCDR). The baselines we compare against have their source code available here (10.1109/TCBB.2019.2919581, DeepDSC) and here: (https://github.com/liuxuan666/SubCDR, SubCDR). We also used Python (v3.8.12), Tensorflow (v2.8.0), Scikit-learn (v1.0.2), and other standard python libraries (e.g. numpy, pandas, etc).

For manuscripts utilizing custom algorithms or software that are central to the research but not yet described in published literature, software must be made available to editors and reviewers. We strongly encourage code deposition in a community repository (e.g. GitHub). See the Nature Portfolio guidelines for submitting code & software for further information.

Data

Policy information about availability of data

All manuscripts must include a data availability statement. This statement should provide the following information, where applicable:

- Accession codes, unique identifiers, or web links for publicly available datasets
- A description of any restrictions on data availability
- For clinical datasets or third party data, please ensure that the statement adheres to our policy

Our manuscript does not produce any new data. The data used in this work is open source and can be accessed as follows: PRISM drug repurposing and drug

genetic target information (version: PRISM Repurposing 19Q4; DOI: https://doi.org/10.1038/s43018-019-0018-6); CCLE cell line gene expression and cancer type classifications (version: DepMap Public 22Q2, DOI: https://doi.org/10.1038/s41586-019-1186-3).

Research involving human participants, their data, or biological material

Policy information about studies with human participants or human data. See also policy information about sex, gender (identity/presentation), and sexual orientation and race, ethnicity and racism.

Reporting on sex and gender	NA
Reporting on race, ethnicity, or other socially relevant groupings	NA
Population characteristics	NA
Recruitment	NA
Ethics oversight	NA

Note that full information on the approval of the study protocol must also be provided in the manuscript.

Field-specific reporting

Please select the one below that is the best fit for your research. If you are not sure, read the appropriate sections before making your selection.

☒ Life sciences ☐ Behavioural & social sciences ☐ Ecological, evolutionary & environmental sciences

For a reference copy of the document with all sections, see nature.com/documents/nr-reporting-summary-flat.pdf

Life sciences study design

All studies must disclose on these points even when the disclosure is negative.

Sample size	The sample sizes of subsets used for training and validating our model were determined by random splitting cell lines stratified by cancer type. A subset of cancer types with a limited number of cell lines (< 15) were withheld as an external test set.
Data exclusions	We exclude cell lines with cancer types labeled as Unknown or Noncancerous
Replication	Training data was split into 5 folds with equal proportions of cell lines from each cancer type such that 5 fold cross validation could be performed.
Randomization	Random splitting of cell lines was stratified by cancer type.
Blinding	Blinding is not relevant for this study as there are no control groups. Furthermore, as long as representative subsets of the data are used for training, validation, and testing, it does not matter to which group each cell line is assigned.

Reporting for specific materials, systems and methods

We require information from authors about some types of materials, experimental systems and methods used in many studies. Here, indicate whether each material, system or method listed is relevant to your study. If you are not sure if a list item applies to your research, read the appropriate section before selecting a response.

Materials & experimental systems

n/a	Involved in the study	
☒	☐	Antibodies
☒	☐	Eukaryotic cell lines
☒	☐	Palaeontology and archaeology
☒	☐	Animals and other organisms
☒	☐	Clinical data
☒	☐	Dual use research of concern
☒	☐	Plants

Methods

n/a	Involved in the study	
☒	☐	ChIP-seq
☒	☐	Flow cytometry
☒	☐	MRI-based neuroimaging

Plants

Seed stocks	NA
Novel plant genotypes	NA
Authentication	NA
